# 526 Keep it up! Inpatient Weight Changes in Burn Patients with >20%TBSA

**DOI:** 10.1093/jbcr/irac012.156

**Published:** 2022-03-23

**Authors:** Tomer Lagziel, Joshua S Yoon, Stephanie L Martinez, Carrie A Cox, Eliana F Duraes, Charles S Hultman, Julie Caffrey

**Affiliations:** Johns Hopkins University School of Medicine / Sackler School of Medicine, New-York Program, Tel-Aviv University, Rockville, Maryland; R. Adams Cowley Shock Trauma Center, Division of Plastic Reconstructive and Maxillofacial Surgery, Herndon, Virginia; Johns Hopkins, Towson, Maryland; Johns Hopkins Bayview Medical Center, Baltimore, Maryland; Johns Hopkins University School of Medicine, Baltimore, Maryland; Johns Hopkins University School of Medicine, Baltimore, Maryland; Johns Hopkins, Baltimore, Maryland

## Abstract

**Introduction:**

Burn patients with >20%TBSA suffer from a hypermetabolic state causing loss of muscle mass as well as a compromised immune system and delayed wound healing. Weight loss is most severe in patients with >20%TBSA with an initial gain of weight due to fluid resuscitation. These findings led the American Burn Association to propose new quality measures for burn-injury admissions, including weight loss from admission to discharge. We aim to assess how our institution’s outcomes adhere to the proposed measures and if our findings correlate with previously described results.

**Methods:**

A retrospective review was conducted for adult patients admitted to our institution with burn injuries of >20%TBSA since 2016. Three groups were established based on %TBSA: 20-29% (Group 1), 30-39% (Group 2), and >40% (Group 3). We assessed weight changes from admission to discharge and performed a multivariate analysis to account for age, sex, number of surgical procedures, and hospital length-of-stay (LOS).

**Results:**

Data from 40 patients suffering burn injuries of >20%TBSA showed 11 patients with %TBSA of 20-29%, 10 patients with %TBSA of 30-39%, and 19 patients with %TBSA of >40%. When comparing groups 1 and 2, we saw significantly more weight loss in group 2 over the course of admission without a significant change in total hospital LOS. The average %weight loss for group 1 was 1.46%, 8.36% for group 2, and 10.56% for group 3. No patients in group 1 had a weight loss >15%. For group 2, patients with weight loss >15% had a significantly longer LOS and underwent significantly more surgical procedures during their admission. For group 3, most patients that experienced weight loss >20% did not have a longer LOS but did require more surgical procedures during their course of admission.

**Conclusions:**

Analysis of the data demonstrates that patients with >20%TBSA do suffer significant weight changes, likely due to extreme metabolic disturbances. Our data suggests that an increased length of stay is not a significant driver for weight loss changes between patients with %TBSA of 20-29 and 30-39, suggesting other pathophysiologic mechanisms in play. Our data supports the idea that patients with %TBSA>40 are a unique subset of patients, requiring specialized nutritional protocols and metabolic analysis.

